# Personalized Feedback on Staff Dose in Fluoroscopy-Guided Interventions: A New Era in Radiation Dose Monitoring

**DOI:** 10.1007/s00270-017-1690-5

**Published:** 2017-05-12

**Authors:** Anna M. Sailer, Laura Vergoossen, Leonie Paulis, Willem H. van Zwam, Marco Das, Joachim E. Wildberger, Cécile R. L. P. N. Jeukens

**Affiliations:** 1 Department of Radiology, Maastricht University Medical Centre, P. Debyelaan 25, 6229 HX Maastricht, The Netherlands; 2 Department of Radiology, Stanford University School of Medicine, 300 Pasteur Drive, Stanford, CA 94303 USA; 3 CARIM School of Cardiovascular Diseases, Maastricht University Medical Centre, 6229 HX Maastricht, The Netherlands

**Keywords:** Endovascular procedures, Radiation exposure, Radiation dosimetry, Radiation monitoring, Occupational dose, Interventional radiology, Radiation protection

## Abstract

**Purpose:**

Radiation safety and protection are a key component of fluoroscopy-guided interventions. We hypothesize that providing weekly personal dose feedback will increase radiation awareness and ultimately will lead to optimized behavior. Therefore, we designed and implemented a personalized feedback of procedure and personal doses for medical staff involved in fluoroscopy-guided interventions.

**Materials and Methods:**

Medical staff (physicians and technicians, *n* = 27) involved in fluoroscopy-guided interventions were equipped with electronic personal dose meters (PDMs). Procedure dose data including the dose area product and effective doses from PDMs were prospectively monitored for each consecutive procedure over an 8-month period (*n* = 1082). A personalized feedback form was designed displaying for each staff individually the personal dose per procedure, as well as relative and cumulative doses. This study consisted of two phases: (1) 1–5th months: Staff did not receive feedback (*n* = 701) and (2) 6–8th months: Staff received weekly individual dose feedback (*n* = 381). An anonymous evaluation was performed on the feedback and occupational dose.

**Results:**

Personalized feedback was scored valuable by 76% of the staff and increased radiation dose awareness for 71%. 57 and 52% reported an increased feeling of occupational safety and changing their behavior because of personalized feedback, respectively. For technicians, the normalized dose was significantly lower in the feedback phase compared to the prefeedback phase: [median (IQR) normalized dose (phase 1) 0.12 (0.04–0.50) µSv/Gy cm^2^ versus (phase 2) 0.08 (0.02–0.24) µSv/Gy cm^2^, *p* = 0.002].

**Conclusion:**

Personalized dose feedback increases radiation awareness and safety and can be provided to staff involved in fluoroscopy-guided interventions.

## Introduction

Radiation safety and protection of patients and medical staff are a key component of medical quality management. A strong trend in medicine toward minimal invasive treatment led to an increase in image-guided interventions [1]. Many of these interventions are performed by radiologists, cardiologists, vascular surgeons and other physicians under fluoroscopy guidance, which carries the risk of radiation-induced tissue reactions and stochastic effects for both patients and health-care professionals [2, 3]. As patients are exposed to the primary X-ray beam, they receive a higher dose during a particular procedure in comparison with physicians and other medical staff members whose exposure mainly originates from radiation scattered from the patient [4–6]. However, the cumulative dose, composed of repetitive exposure to scattered radiation from fluoroscopy-guided procedures performed, can add up to a substantial individual staff member’s work-related radiation burden [7, 8]. Patient dose monitoring systems are recommended by national and international advisory boards [9, 10], and comprehensive dose registration will be obligatory in Europe in the near future [11]. Physicians and technicians play an essential role in the safe use of fluoroscopy in medical practice. Appropriate use of interventional imaging techniques (e.g., fluoroscopy, digital subtraction angiography (DSA), road map and cone-beam computed tomography (CBCT)) requires knowledge of their potentially harmful effects on both patients and staff [1]. We hypothesize that providing medical staff with short term, i.e., weekly, personal feedback, containing both patient and staff doses, will increase awareness and leads to optimized behavior. As such, it may be considered as a new approach to dose optimization for both patients and staff members. Aim of the study was to design, implement and assess a personalized feedback of patient and staff doses for medical staff involved in fluoroscopy-guided interventions.

## Materials and Methods

The study was approved by the institutional ethical committee. Employees enrolled in this study gave their written informed consent. Written informed consent of the patients involved in the procedures was waived.

### Data Collection

An automated patient and staff dose tracking system (DoseWise Portal, Philips Healthcare, Best, The Netherlands) was installed in our radiology angiosuite and hybrid operating room (Philips Allura Xper, Philips, The Netherlands) in October 2015. With this new system, data such as the type of procedure and total dose area product (DAP), as well as single X-ray event-related data, such as the type of X-ray technique (digital subtraction angiography, fluoroscopy, road map or 3D acquisitions) and corresponding DAP, were recorded. Furthermore, the data were simultaneously linked to real-time staff dose measurements for the complete procedure as well as for all separate X-ray acquisitions within a procedure. For this purpose, all team members (radiologists, endovascular surgeons (*n* = 9) and radiology technicians (*n* = 18), in total *n* = 27) involved in fluoroscopy-guided interventions were equipped with personal dose meters (PDMs, DoseAware, Philips, The Netherlands). PDMs were stored overnight in a metal rack, and employees were encouraged to wear them during the procedures outside the lead apron on the left breast pocket. A reference PDM was mounted on the C-arm to obtain a reference measure of the scattered dose at a fixed distance without any additional radiation protection measures. The PDMs were calibrated to measure the personal dose equivalent Hp(10), which is an internationally acknowledged representative for effective dose [12] in case no additional protective garments are worn. For all procedures, the attending physicians and technicians and their role in the individual procedures, as well as the used radiation protection shielding (standard table curtain, additional table-side shield, additional ceiling-mounted shield), were prospectively recorded in an in-house developed digital database. Data were prospectively collected for each consecutive procedure for eight consecutive months (November 2015–June 2016; *n* = 1082).

### Study Design

A live monitor, which was installed next to the main screen in the angiosuite and hybrid OR in 2013, displayed the current dose rate from the PDMs and was visible during all procedures. This study consisted of two phases: (1) months 1–5: staff not receiving personalized dose feedback (*n* = 701) and dose data for each procedure were prospectively collected and (2) month 6–8: staff receiving weekly individual personalized dose feedback (*n* = 381). After eight months, the dose feedback was evaluated anonymously through questionnaires.

### Personalized Dose Feedback

**Fig. 1:**
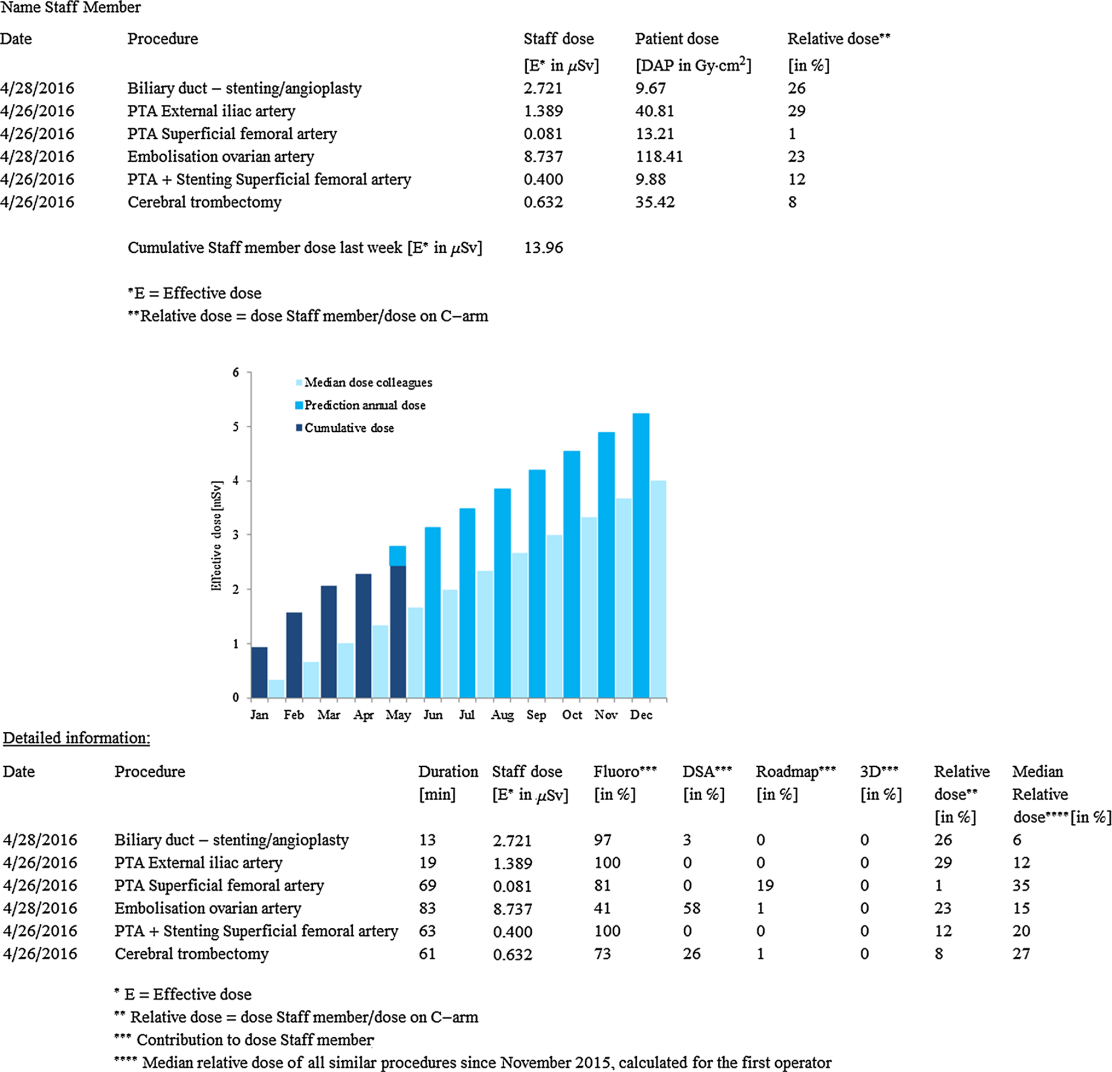
Example of a weekly personalized dose feedback form of a physician

A personalized feedback form was designed displaying for each employee individually the patient dose (DAP) and staff personal effective dose (E) per procedure they were involved in, as well as the relative dose (staff PDM dose/reference PDM dose × 100%). An anonymous comparison to the median operator relative dose of each procedure type was also provided. In addition, a graph showed the cumulative dose received the current year until the date of the feedback and an extrapolation to the estimated expected annual dose. Detailed dose data showing the contribution from different types of acquisitions (fluoroscopy, DSA, road map and 3D acquisitions) concluded the feedback form. Feedback forms were generated semiautomatically by means of in-house developed software program (Mathematica version 10.2, Wolfram Research Inc., Champaign, Illinois) and were sent out to all employees individually by email on a weekly basis. An example on a weekly personalized feedback form is shown in Fig. 1.

### Assessment of Personalized Dose Feedback

Wearing of the individual PDMs by employees before and during feedback was evaluated as follows: The attending physicians and technicians are registered in the in-house database. By comparing these to the PDMs automatically registered by the DoseWise Portal for each procedure, the average percentage of personnel wearing the PDMs was determined for the two phases.

Physician and technician doses were compared between phase 1 (without feedback) and phase 2 (with feedback). As individual employees in an academic center are involved in procedures in different roles which introduce an extra variability, we performed this analysis based on the role first operators (FO) and first technicians (FT). All procedures were selected by a radiation research fellow for which these roles could be clearly identified from the in-house database (phase 1: *n* = 369 out of 701, phase 2: *n* = 200 out of 381). In addition, the use of radiation protection tools, namely table drape, table shield and ceiling shield, in phases 1 and 2 was evaluated.

**Fig. 2:**
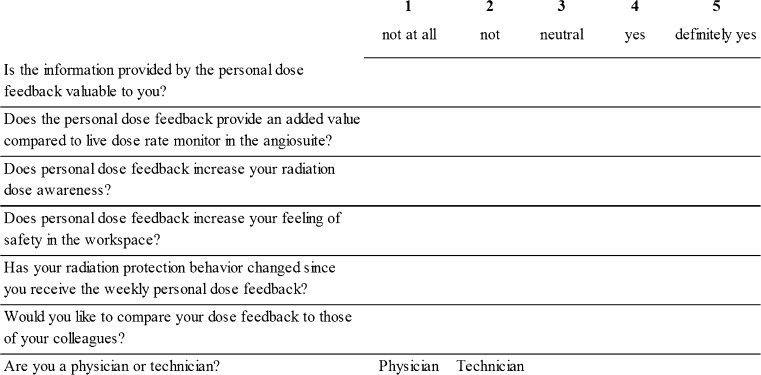
Closed questions of the questionnaire to evaluate the personalized dose feedback among medical staff

Subjective assessment of personalized dose feedback was performed by means of questionnaires, which were sent to the medical staff 3 months after implementation of the personalized feedback. Questions included closed questions with prescribed answered scoring from 1 to 5 (Fig. 2) as well as additional open questions. Closed questions were used for evaluation of the feedback. Questionnaires were collected and analyzed anonymously, with the exception of an assessment of physician versus technician.

### Statistical Analysis

Personal doses (E) were analyzed after normalization to the corresponding procedure dose (DAP) in order to correct for variation in total radiation output between individual procedures in pre- and postfeedback phases. Normalized staff doses were tested for normal distribution; differences in normalized staff dose before and during the feedback phase were tested for statistical significance for FO (*E*_FO_/DAP) and FT (*E*_FT_/DAP) separately using Mann–Whitney *U* test where applicable (SPSS statistics 20.0, Chicago, Illinois). Employees’ answers to the questionnaires were displayed graphically. Two-sided *p* values <0.05 were considered significant. Questionnaire responses were not correlated with staff dose due to an anonymous evaluation of the questionnaires.

## Results

In phase 2 (with dose personalized feedback), the wearing of PDMs by physicians and technicians increased by 13%, from 75 to 88% compared to phase 1 (without feedback). Distribution of the staff and procedure doses before and after feedback is shown in Table 1. For the first technician (FT), the normalized dose was significantly lower in the feedback phase compared to the prefeedback phase (median (IQR) relative normalized FT dose: 0.12 (0.04–0.50) versus 0.08 (0.02–0.24) µSv/Gy cm^2^, *p* = 0.002). The normalized first operator (physician) doses showed no significant difference before and during the feedback; median (IQR) normalized FO dose: 0.52 (0.17–1.45) µSv/Gy cm^2^ (phase 1) versus 0.40 (0.15–1.27) µSv/Gy cm^2^ (phase 2), *p* = 0.24. The use of the radiation protection tools table drape, table shield and ceiling shield was increased by 2, 15 and 28% during the feedback phase, respectively.

**Table 1:** Distribution of staff doses during phase 1 (pre feedback) and phase 2 (with feedback)

			Median	25 Percentile	75 Percentile	Mean	SD	Min	Max
Normalized dose FO (E/DAP)	Prefeedback	[μSv/Gycm²]	0.52	0.17	1.45	<0.01	11.73	1.19	1.81
	With feedback	[μSv/Gycm²]	0.4	0.15	1.27	<0.01	6.82	0.95	1.32
Normalized dose FT (E/DAP)	Prefeedback	[μSv/Gycm²]	0.12	0.04	0.5	<0.01	13.2	0.6	1.42
	With feedback	[μSv/Gycm²]	0.08	0.02	0.24	<0.01	10.37	0.32	0.95
Absolute over-lead dose FO (E)	Prefeedback	[μSv]	12.11	2.12	33.11	0.02	614.1	37.05	73.11
	With feedback	[μSv]	11.68	3.11	35.91	<0.01	603.92	37.17	77.54
Absolute over-lead dose FT (E)	Prefeedback	[μSv]	2.64	0.76	5.91	<0.01	136.92	6.45	14.04
	With feedback	[μSv]	2.05	0.767	5.49	<0.01	547	9.7	50.07

*E* effective dose. *DAP* dose area product. *SD* standard deviation

Staff doses presented as normalized for procedure dose as well as absolute values

### Evaluation of Questionnaires

**Fig. 3:**
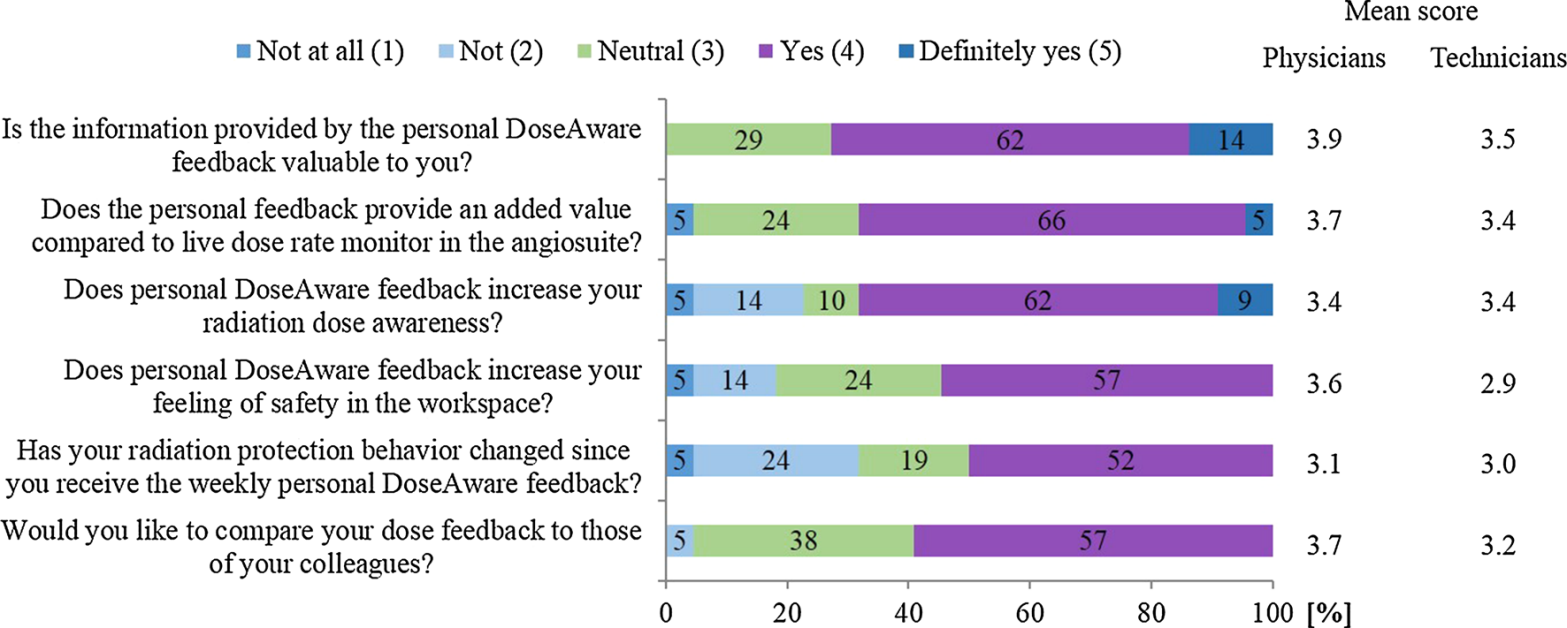
Graphical representation of scores of medical staff’ answers on the questionnaire’s closed questions. Numbers in colored bars indicate percentages

The response rate on the feedback questionnaire was 78% (21/27 returned questionnaires, 8 physicians and 13 technicians). In Fig. 3, the distribution of answers to the closed questions in the questionnaire is shown. The individual dose feedback was scored as valuable by 76% of the respondents; there was no difference in average scoring between physicians and technicians (*p* > 0.05). 71% of the team members reported that the feedback increased their personal radiation dose awareness, and 57% answered that the feedback increased their feeling of occupational safety or even had changed their behavior (52%).

## Discussion

Quality and safety management play a key role in human medicine. Ionizing radiation carries the risk of radiation-induced tissue reactions and stochastic effects for both patients and medical staff [13]. Patients’ benefit from minimally invasive interventional procedures is indisputable [14]. While patient dose is justified by medical indication, radiation exposure for health-care professionals has to be monitored even more carefully due to its repetitive character. Medical staff working with ionizing radiation should be aware of the radiation dose they and their patients may receive during a particular procedure and which factors determine the level of these doses [15, 16]. Knowledge on personal and procedure radiation dose based on individual dosimetry and personalized feedback allows for optimal use of the ALARA principles [17].

The present study introduces a new concept of personalized weekly feedback of patient and staff doses to medical staff as an integral part of the clinical workflow. Previous work from the ORAMED project (Optimization of RAdiation protection for MEDical staff) has shown that doses received by physicians depend heavily on individual practice [18, 19]. Personalized feedback is a next step in radiation dose monitoring and aims to enhance knowledge and stimulate intrinsic motivation of medical staff to optimize procedure and personal doses. Implementation of individual dose monitoring and weekly personalized dose feedback proved technically feasible by means of an automated combined patient and medical staff dose monitoring system and semiautomated feedback generation. In general, the staff wore a lead apron, thyroid collar and sometimes leaded glasses, so the over-lead dose measurements are an overestimation of the actually received effective staff dose. Nevertheless, unshielded body parts such as the extremities and (often) the lens of the eye are not protected when directly exposed to the scattered radiation [18]. As the Dutch legal dosimetry is reported back to the staff as over-lead Hp(10) values, the same measure was presented in the feedback forms as the staff is familiar in interpreting these values. To increase awareness and to maximize the educational effect, the feedback was presented within a short time span after performing the procedures. The medical staff indeed indicated that awareness for radiation exposure was increased and a positive behavioral change with respect to radiation safety was experienced. Moreover, the results show that personal over-lead doses decreased significantly for technicians during the feedback phase, whereas the median absolute and normalized FO dose displayed a nonsignificant trend toward dose reduction. This difference might be due to the fact that technicians have more options to seek distance during X-ray exposure than physicians. Although the absolute and normalized technician doses were low compared to the physician doses, the personalized feedback resulted in significant dose reduction. To set the FO doses in perspective, the median over-lead FO doses of roughly 12 µSv per procedure (pre- and postfeedback) are about 2000 times lower than the annual legal dose limit of 20 mSv for interventional radiologists in Europe [11]. However, the large range and maximum doses of >600 µSv indicate that awareness by interventional radiologists of such occasional ‘high personal dose procedures’ is necessary. As the nature of our questionnaire was anonymous, no correlations could be deduced between positive/negative answers in the questionnaire and an individual decrease/increase in personal dose. Further research has to be performed to evaluate long-term effects of feedback on medical staff dose with regard to individual responsiveness to personalized dose feedback. Such an evaluation could provide further insights into improvement in personalized feedback and, in general, how to promote radiation safety.

Real-time, in-room dose feedback to medical staff may also raise awareness of high exposure [20]. Previous studies have shown positive effects on occupational doses of monitors that provided real-time feedback on radiation exposure, either visually [21–26] or auditory [27]. From our experience, a disadvantage of the visual monitor is that in particular the first operator cannot constantly keep track of the screen as his/her attention needs to be focused on the procedure. Furthermore, real-time feedback provides momentary dose rate information during an individual procedure only. However, retrospective procedure dose information in particular in comparison with similar procedures allows for more reflection. Our results indeed demonstrated that the personalized feedback is an effective radiation awareness tool in addition to live monitoring, which was already used in clinical practice in our center. In this sense, personalized feedback can be regarded as a staff dose optimization tool induced by a behavioral change resulting from increased awareness, rather than optimizing protocols or introducing new dose reduction techniques.

## Limitations

There are limitations of the current feedback system. Firstly, it requires the acquisition of electronic PDMs for all team members. The costs of implementation could, however, reduce significantly if team members were able to share PDMs between procedures and connect to the system with the PDM for each procedure separately. Secondly, the weekly feedback was generated semiautomatically, which was time-consuming (2 h per week), and could therefore only be provided at a weekly interval. Implementation of an automated feedback form is currently in development. For this, a Web-based implementation will allow staff to individually log on to the system and receive their personal feedback on demand, even directly after each procedure.

If broadly adapted, dose values obtained from combined patient and staff monitoring and implemented in an automated dose software could be used for general dose analysis. This could be used for quality improvement in radiation shielding tools in order to achieve optimization of boundary conditions that determine occupational and patient safety such as procedure setup, or in-room and personal radiation protection tools. Ultimately, these data can be used for benchmarking and knowledge transfer of procedure doses among institutes, thereby promoting optimization of radiation protection, boundary conditions and individual behavior.

## Conclusions

Patient dose and medical staff effective doses from personal dose meters can be monitored simultaneously by an automated real-time dose tracking system and can be used to create personalized feedback on occupational and patient radiation dose. Personalized dose feedback is able to increase health-care professionals’ radiation awareness as well as to improve radiation safety and individual protection in the clinical setting. Personalized dose feedback can be used as a dose optimization tool and for benchmarking of patient and staff doses, while educating staff and initiating a change in behavior.
